# Claims of the Temperature Reformation

**Published:** 1846-03

**Authors:** 


*Claims of the Temperance Reformation.*—An uncommonly dignified and persuasive address to the people of Massachusetts, on the present condition and claims of the temperance reformation, has recently been published by the association known as the Massachusetts Temperance Union. Seeing upon the title page the names of the President of Williams College, Hon. Samuel Hoar, and Dr. Woodward, of Worcester, we were induced to give more than ordinary attention to the address. It emanates from a high source, and cannot fail to command the respect of all men who love order, health and happiness. No class of persons understand the necessity of temperance better than physicians, and we feel quite sure that their untiring and unflinching efforts will always be in favor of the cause that is doing so much for the moral and physical reformation of those who have loved strong drink.—*Ibid.*

